# Effects of Climate Variability on Malaria Transmission in Southern Côte d’Ivoire, West Africa

**DOI:** 10.3390/ijerph20237102

**Published:** 2023-11-23

**Authors:** Madina Doumbia, Jean Tenena Coulibaly, Dieudonné Kigbafori Silué, Guéladio Cissé, Jacques-André N’Dione, Brama Koné

**Affiliations:** 1 Unité de Formation et de Recherche des Sciences Biologiques, Université Péléforo Gon Coulibaly, Korhogo BP 1328, Côte d’Ivoire; brama.kone@csrs.ci; 2 Centre Suisse de Recherches Scientifiques en Côte d’Ivoire (CSRS), Abidjan 01 BP 1303, Côte d’Ivoire; jean.coulibaly@csrs.ci (J.T.C.); kigbafori.silue@csrs.ci (D.K.S.); 3 Unité de Formation et de Recherche Biosciences, Université Félix Houphouët-Boigny, Abidjan 22 BP 582, Côte d’Ivoire; 4 Department of Epidemiology and Public Health, Swiss Tropical and Public Health Institute, CH 4002 Basel, Switzerland; gueladio.cisse@swisstph.ch; 5 Faculty of Science, University of Basel, CH-4003 Basel, Switzerland; 6 Centre de Suivi Ecologique, BP 15532, Fann Résidense, Dakar 10700, Senegal; jacandrendione@yahoo.fr

**Keywords:** malaria, climate, VECTRI, EIR, vector density, Tiassalé

## Abstract

Malaria continues to be a major public health concern with a substantial burden in Africa. Even though it has been widely demonstrated that malaria transmission is climate-driven, there have been very few studies assessing the relationship between climate variables and malaria transmission in Côte d’Ivoire. We used the VECTRI model to predict malaria transmission in southern Côte d’Ivoire. First, we tested the suitability of VECTRI in modeling malaria transmission using ERA5 temperature data and ARC2 rainfall data. We then used the projected climatic data pertaining to 2030, 2050, and 2080 from a set of 14 simulations from the CORDEX-Africa database to compute VECTRI outputs. The entomological inoculation rate (EIR) from the VECTRI model was well correlated with the observed malaria cases from 2010 to 2019, including the peaks of malaria cases and the EIR. However, the correlation between the two parameters was not statistically significant. The VECTRI model predicted an increase in malaria transmissions in both scenarios (RCP8.5 and RCP4.5) for the time period 2030 to 2080. The monthly EIR for RCP8.5 was very high (1.74 to 1131.71 bites/person) compared to RCP4.5 (0.48 to 908 bites/person). These findings call for greater efforts to control malaria that take into account the impact of climatic factors.

## 1. Introduction

Malaria is the leading cause of hospital visits in Côte d’Ivoire, with more than 40 to 62% of hospitalizations occurring among children less than 5 years of age [1,2]. In most endemic countries, children under five years old and pregnant women are the most vulnerable and are therefore more likely to develop severe malaria [3]. In 2017, the estimated number of malaria cases and deaths was approximately 3.5 million and 10 thousand in Côte d’Ivoire, respectively [4]. Pregnant women are the most affected. Indeed, the risk of death from severe malaria is twice as high in pregnant women [5]. This is due to the fact that pregnancy decreases immunity and increases the likelihood of severe anemia [6].

The transmission of malaria in Côte d’Ivoire is primarily carried out by five main species of mosquito, namely, *An. Gambiae s.s.*, *An. Coluzzii*, *An. funestus s.s*., *An. nili s.s.*, and *An. Melas*, with the first three being the most predominant species in transmission. However, *An. nili s.s.* and *An. melas* can play important roles in certain areas of the country [7,8]. These vector species are therefore not homogeneously distributed, and their vectorial capacity also varies enormously depending on the location and the season [7,9,10,11], leading to heterogeneity in the transmission rate across the country [3]. Of note, human activities, through the establishment of rice fields in some regions, provide additional optimal conditions for Anopheles mosquito breeding and larval development [9,12,13]. In addition, the impact of climatic variables on malaria varies across the region and the ecological systems [14,15]. Numerous studies have used statistical malaria models, such as the regression model, the Bayesian model, and the Hydrology, Entomology, and Malaria Transmission Simulator (HYDREMATS), to determine the effect of climate change on reported malaria rates in endemic malaria regions of Africa [16,17,18,19,20,21,22]. In the northern part of Côte d’Ivoire, for example, M’Bra et al. [16] have demonstrated that an incremental increase of 10 mm of monthly precipitation was, on average, associated with a 1% and a 1.2% increase in the number of clinical malaria episodes with 1 to 2 months lag by using negative binomial regression models. These models cannot reproduce well the vector population density, the entomological inoculation rate (EIR), the force of infection, or the infection prevalence or parasite rate (PR) [23,24,25]. In contrast to other models, the vector-borne disease community model VECTRI was first used to highlight the impact of climate variability and change on malaria transmission in West Africa, mainly in Burkina Faso, Cameroon, Ghana, and Senegal [21,26,27,28,29,30,31,32,33]. The VECTRI model reflects how climatic variables affect the biology of the vector and the parasite and therefore the transmission of the disease. The benefit of the VECTRI model is its calibration considering hydrology, evaporation, infiltration, weather, and population migration. The VECTRI model is a dynamic biological model, in contrast to the simple statistical approaches [31]. The VECTRI model is also able to simulate EIR, vector density, malaria cases, etc. Hence, it appears to be the best tool for assessing the effect of climate variability on malaria transmission.

Our study aims to enhance the understanding of rainfall and temperature effects on the transmission of malaria in the southern part of Côte d’Ivoire, characterized by an equatorial transition climate [34], by using the VECTRI model. Although several studies have been published that have assessed the relationship between climatic parameters and malaria transmission using the VECTRI model, the potential of this model remains unexplored in Côte d’Ivoire. The key findings of the current study could be considered in the national malaria control strategies to support the ongoing effort of elimination.

## 2. Materials and Methods

### 2.1. Ethics Statement

The Climate Research for Development (CR4D) via the African Academy of Sciences granted this research project. The study was approved by the Comité National d’Éthique des Sciences de la Vie et de la Santé of Côte d’Ivoire (N/Ref: 114-20/MSHP/CNESVS_km, dated 17 August 2020). The local Health District Officer’s permission was also obtained. The participants were enrolled, and signed consent was required for adults, in addition to assent for participants below 18 years. Clinical malaria cases were treated according to the national policy for the management of malaria cases.

### 2.2. Study Area

**Figure 1:**
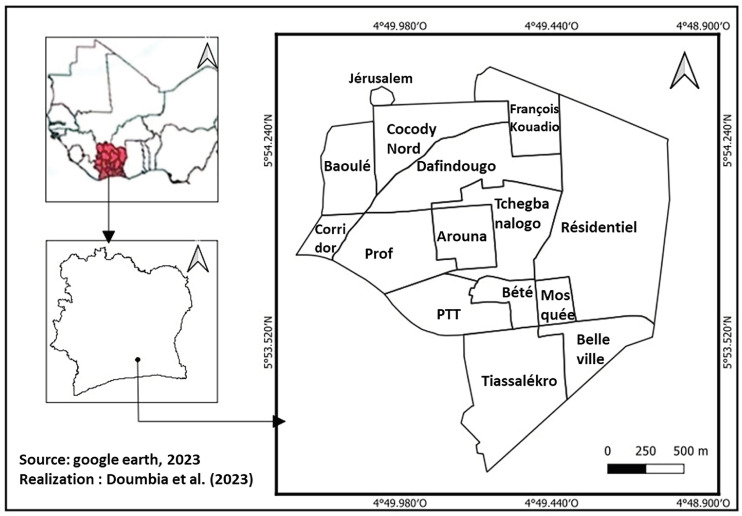
Map of the study area (Tiassalé) located in the southern part of Côte d’Ivoire (5°53′ N, 4°49′ W).

The health district of Tiassalé is located in the southern part of Côte d’Ivoire (5°53′ N, 4°49′ W), in the tropical zone (Figure 1). It is characterized by an equatorial transition climate, governed by the meridional movement of the intertropical discontinuity or convergence zone.

The annual rainfall in Tiassalé ranges between 600 and 2000 mm per year, with four distinct climatic seasons that are (1) a long rainy season from March to June, (2) a short dry season from July to August, (3) a short rainy season from September to November, and (4) a long dry season from December to March. Two-thirds of the annual rainfall is observed during the long rainy season. The city of Tiassalé is part of the evergreen forest zone of the country, which includes flooded swampy forest and hydromorphic soil.

Furthermore, the city and its surroundings are characterized by rice cultivation, which has led to the development of mosquito breeding sites [35]. The city’s annual relative humidity is very high, with an average of 90%. The average duration of sunshine is around 2000 h. The population of Tiassalé has increased from 26,580 inhabitants in 2010 to 83,648 in 2021, with an estimated growth rate of 11.91% [35,36]. The population density has increased from 30 inhab/ha in 1992 to 118.2 inhab/ha in 2021 [35,37].

### 2.3. Study Design

#### 2.3.1. Data Collection

##### Malaria Data

Data on malaria cases were obtained from health facilities using the patient consultation register and extracted by trained health workers (nurses) recruited from the Tiassalé health district. As described elsewhere [16], a malaria case refers to an individual who exhibits fever symptoms or fever accompanied by headaches, back pain, chills, sweats, myalgia, nausea, or vomiting and who has been clinically diagnosed, with an additional confirmed laboratory test consisting of a rapid diagnostic test plus blood smears.

##### Meteorological Data

There is currently no synoptic weather station located in Tiassalé. We consequently used satellite data covering the period 1987 to 2019 from Africa Rainfall Climatology version 2 (ARC2), which was obtained from the European Organization for the Exploitation of Meteorological Satellites (EUMETSAT), and from the European Center for Medium-Range Weather Forecasts (ECMWF) reanalysis v5 (ERA5). We also used temperature and rainfall data from four weather stations surrounding Tiassalé and located in the cities of Abidjan, Yamoussoukro, Gagnoa, and Dimbokro to assess the accuracy of the ERA5 and ARC2 data. The observational data of the four stations were from 1960 to 2019 and were provided by the *Société D’exploitation et de Développement Aéroportuaire, Aéronautique Et Météorologue* (SODEXAM), which is responsible for meteorological data collection, analysis, and management in Côte d’Ivoire.

##### Data from the ERA5 Reanalysis Database

ERA5 Reanalysis is the fifth-generation reanalysis product from the European Centre for Medium-Range Weather Forecasts (ECMWF) for the global climate. It provides hourly data at a high spatial resolution available at 10 Km, based on statistical and/or remote sensing for many land applications [38,39,40,41], and combines vast amounts of historical observations into global estimations using advanced modeling and data assimilation systems. The data produced in ERA5 are most valuable as substitutes for observational data where such data are limited or unavailable [33]. The 2 m surface air temperatures of ERA5 are similar to those of in situ observations in some studies, although some bias has been observed in some others [33,42,43]. We used the 2 m air daily maximum (T_max_) and daily minimum temperatures (T_min_).

##### African Rainfall Climatology Version 2 (ARC2)

ARC2 is a revision of the first version of the ARC and uses inputs from two sources: (i) 3-hourly geostationary infrared (IR) data centered over Africa from the European Organization for the Exploitation of Meteorological Satellites (EUMETSAT) and (ii) quality-controlled Global Telecommunication System (GTS) gauge observations reporting 24 h rainfall accumulations over Africa. ARC2 is expected to provide users with real-time monitoring of the daily evolution of precipitation, which will be instrumental in improving decision making. The ARC2 database has been available since 1983, a longer period than the Rainfall Estimate, which also justifies the choice of ARC2 for precipitation in Tiassalé (1987 to 2019).

##### Model Input Data from CORDEX

We used data from the climate change scenario based on a set of 14 simulations (Figure A5) from the most up-to-date ensemble of high-resolution regional climate model (RCM) projections produced in recent years for Africa from the Coordinated Regional Climate Downscaling Experiment (CORDEX) Africa [44,45]. In this ensemble, the simulation period is 1976–2100 over Africa (24.64° W–60.28° E, 45.76° S–42.24° N), with a spatial resolution of 0.44° (~50 km) in latitude and longitude under the Representative Concentration Pathways (RCPs) for climate stabilization (RCP4.5) and high greenhouse gas emissions (RCP8.5). These RCP scenarios correspond to radiative forcings of about 4.5 w/m^2^ and 8.5 w/m^2^ generated by an anthropogenic production of about 660 eq-CO_2_ and 1370 eq-CO_2_, respectively.

#### 2.3.2. Data Analysis

- Assessing the suitability of CORDEX Models in generating climate projections for the Tiassalé region

**Figure A4:**
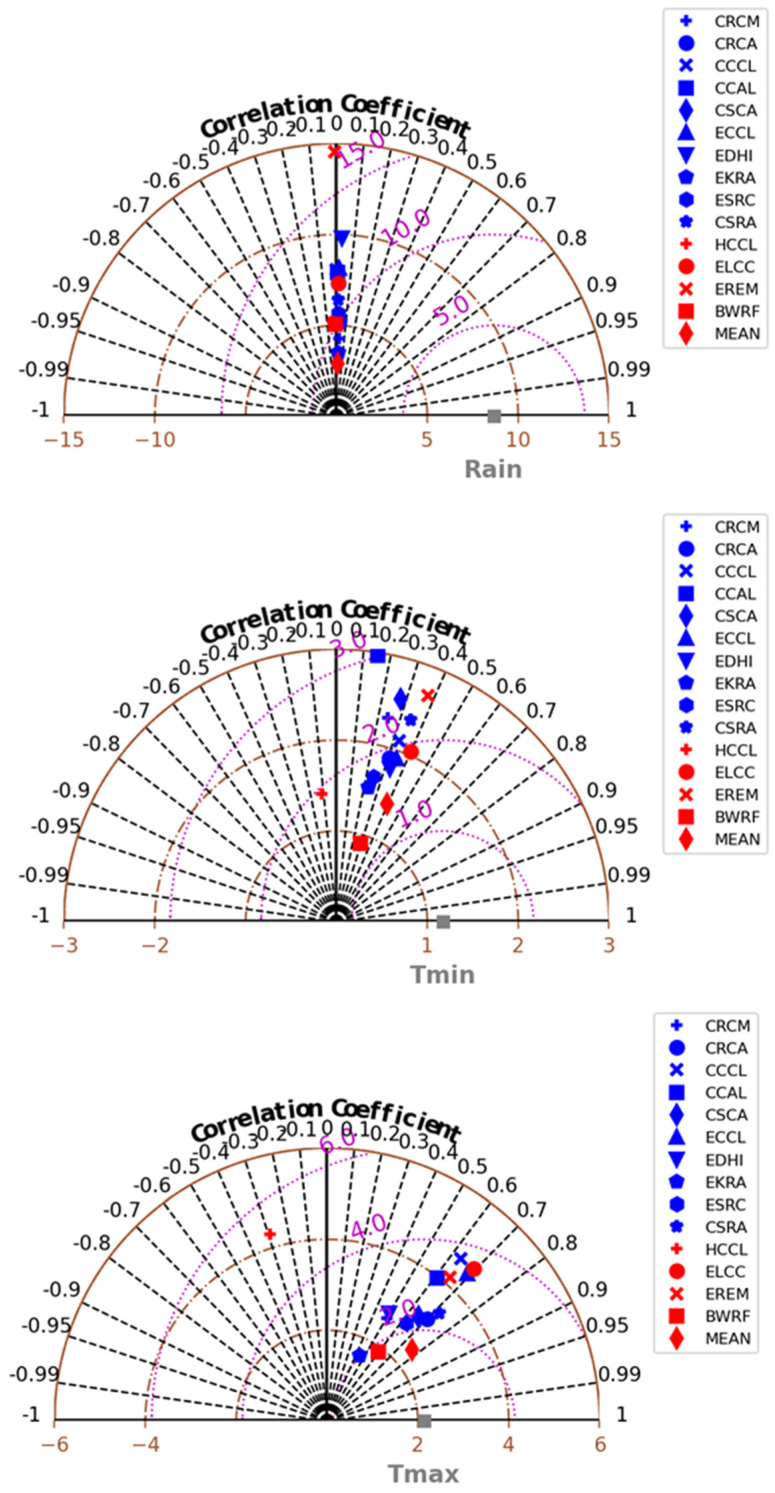
Taylor diagrams obtained from the 14 models of CORDEX, model-averaged and observation data for Tiassalé. The dotted lines indicate the correlation coefficient between models and the reference dataset; the circles indicate standard deviations (blue) and root mean square errors (magenta).

**Figure A5:**
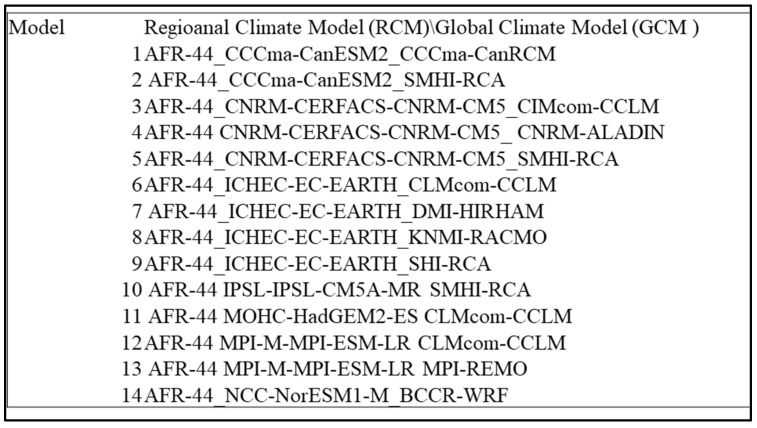
List of 14 simulations taken from the CORDEX-AFRICA data. In this ensemble, each experiment comprises the historical and scenario (RCP4.5 and RCP8.5) runs.

Taylor diagrams [46] were used in the CORDEX model intercomparison with observed data to identify the best-performing model. The evaluation was applied to historical data from ERA5 and ARC2 and observational data (Figure A4). In addition, we used RCP4.5 and RCP 8.5, although the latter represents the most realistic warming scenario considering today’s global greenhouse gas emission trajectory [47,48] and has been widely used for the analysis of projections in sub-Saharan Africa [49,50,51,52]. Prediction data from CORDEX models were collected pertaining to 2030, 2050, and 2081. We used a multi-model mean (EMM) of the CORDEX-Africa simulation scenario, which is currently the most accurate one. Then, the prediction data from CORDEX were corrected using cumulative distribution function transform (CDFt) bias-corrected simulations, as described elsewhere [53,54,55]. The data were corrected based on different calibration periods and were compared with observational data (ERA5 and ARC2) to correct the bias of the historical data from Cordex. Then, these corrected historical data from Cordex were used to carry out the projection (2030–2080) for the RCPs. Whatever the calibration period used, CDFt corrected well the mean state of variables and preserved their trends, as well as daily rainfall occurrence and intensity distributions [54].

b. Malaria morbidity

The monthly and annual values for the number of malaria cases were computed and used for interannual and monthly analysis with the seasonal and annual distribution of precipitation and temperature.

c. Using the VECTRI model to predict malaria transmission in the health district of Tiassalé

**Figure A6:**
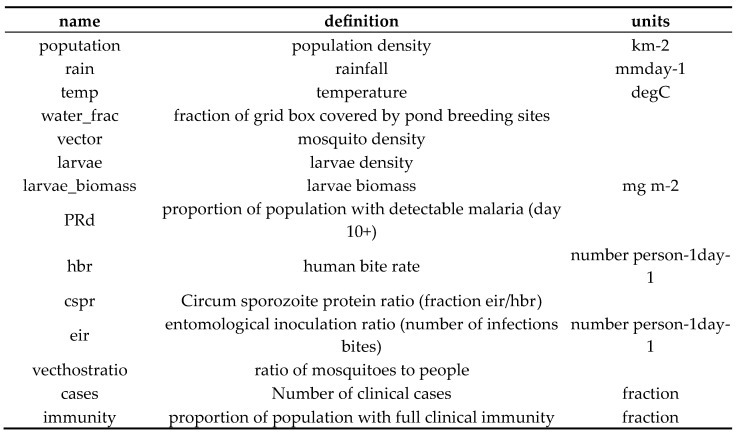
The VECTRI outputs (some of these are optional and switched off by default).

VECTRI model Version v1.3.0 was developed in the Abdus Salam International Centre for Theoretical Physics (ICTP) in Trieste (Italy) by Tompkins and Ermert [26]. VECTRI is a piece of software focused on mathematical models dealing with malaria that accounts for the effects of daily temperature and precipitation on *P. falciparum* and *An. gambiae* life cycles [26] to investigate malaria transmission patterns. VECTRI accounts for the effects of temperature on the sporogonic and gonotrophic cycles, as well as the mortality rate, of adult mosquitoes. We used the default parameters of the model with the new surface hydrology scheme [26,26]. After running the model, the simulated output variables (Figure A6) considered in this study were the adult mosquito (vector) density (per square meter) and the entomological inoculation rate (EIR) corresponding to the number of infective bites per person per day, because they can establish a direct link between malaria transmission and climatic variables. Also, the VECTRI model was able to predict EIR values that were compared by monthly variations in reported malaria cases from Kumasi in Ghana [27]. We then computed the correlation coefficient between the VECTRI output (EIR) and the malaria case data obtained from health registers. For the VECTRI simulations, we used daily rainfall (mm day^−1^), T_min_ and T_max_ (°C), and population density (per square meter) in Tiassalé between 1987 and 2019. Assessing the suitability of ARC2 and ERA5 data was used to back their choice in the meteorological data collection (Appendix A). In Tiassalé, the human population density used for the simulation was 0.53 inhabitants per Km^2^ [35]. To simulate transmission at horizons 2030, 2050, and 2081 for the RCP4.5 and RCP8.5 scenarios, we used the prediction data of rainfall and temperature from CORDEX-Africa, corrected using CDFt bias-corrected simulations [53,54,55]. The predicted values were used in VECTRI to simulate transmission patterns (using EIR and vector density) at the same time horizons for both scenarios in Tiassalé.

## 3. Results and Discussion

### 3.1. Temporal Distribution of Malaria Cases in Tiassalé

**Figure 2:**
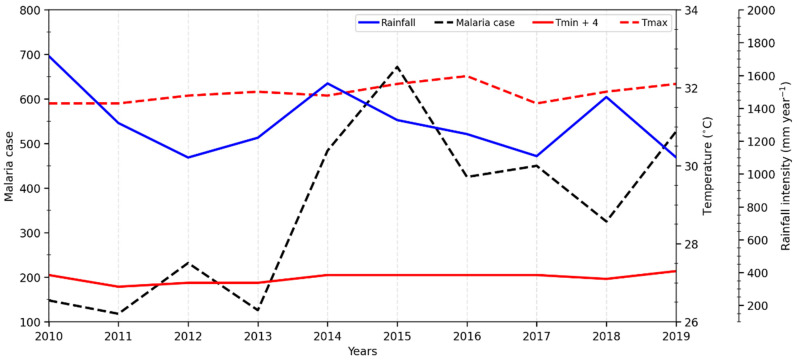
The interannual dynamic of climatic factors and malaria cases in Tiassalé from 2010 to 2019. (T_min_ + 4 was used in order to have the same scale as T_max_.)

Figure 2 shows the interannual distribution of climatic factors and malaria cases between 2010 and 2019 in Tiassalé. A strong increase in the number of malaria cases in Tiassalé was observed, starting in 2014. This increase could be linked to the significant rate of population growth (10.4% from 2010 to 2014) in this malaria-endemic area, which could explain the rise in the number of malaria cases.

Even if the number of malaria cases was permanently high over the years, we found that it varied greatly over the years, with three important peaks observed in 2015, 2017, and 2019, whereas the lower malaria case rates in 2011 and 2013 coincided with years of low to moderate rainfall and high T_max_.

**Figure 3:**
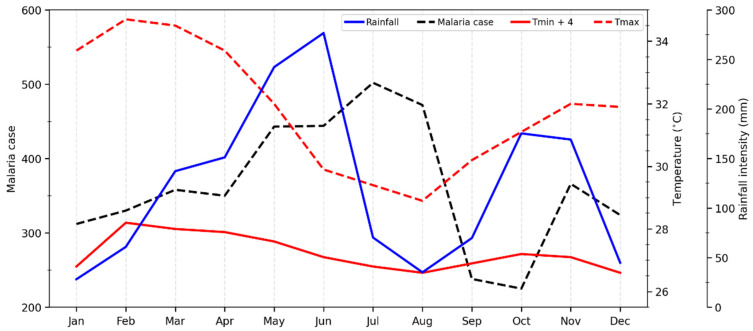
The monthly variation in climatic factors and malaria cases for the period of 2010–2019. (T_min_ + 4 was used in order to have the same scale as T_max_ in the figure.)

Figure 3 shows the intra-annual variability of monthly mean values for rainfall, T_min_, T_max_, and malaria cases from 2010 and 2019.

The peaks for rainfall were in June, October, and November, while malaria case peaks were in July, October, and November. Rainfall and malaria case rates were lower in August and September, respectively. Malaria transmission was low over two months (September and October), and a one-month lag was observed between the peak rainfall and the number of malaria cases. The results clearly show that peak malaria transmission generally follows rainfall.

The monthly pattern of malaria observed in Tiassalé, southern Côte d’Ivoire, is distinct from that observed in Korhogo, in northern Côte d’Ivoire, in the study of Mbra et al. [16]. Of note, Tiassalé and Korhogo belong to two climatic zones that differ in their rainfall regimes [56,57,58] and are located in forest and savannah, respectively. M’Bra et al. [16] showed that, in Korhogo, a high number of malaria cases were recorded at the beginning of June, one month after the beginning of the rainy season, starting in May until November, 2 months after the end of the rainy season. Conversely, the lowest numbers of malaria cases were observed between December and April (during the dry season). However, in Tiassalé, malaria cases have been high over the years and follow the same trend as the forest zone in Ghana, the neighboring country, with low transmission only occurring over three months between February and April [27].

### 3.2. Suitability of the VECTRI Model for Predicting Malaria Transmission in Tiassalé

**Figure 4:**
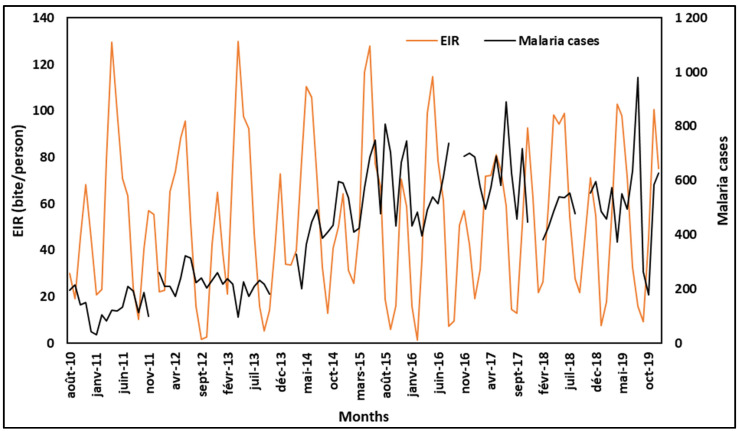
Monthly malaria cases in Tiassalé from 2010 to 2019 and the VECTRI-simulated entomological inoculation rate (EIR).

Figure 4 shows the monthly variation of the VECTRI-simulated EIR and malaria cases in Tiassalé from 2010 to 2019. The patterns of malaria based on observed malaria case data and the EIR simulated with the VECTRI model are globally similar, despite the disparity in the magnitude of each pattern from one month to another, leading to a weak correlation coefficient (Table A2). The slight differences between the EIR and malaria cases may be due to the fact that the VECTRI results are based on 32-year climatology simulations, while malaria cases are reported values. Despite this, VECTRI has demonstrated its ability to simulate malaria patterns, and it has already been used across some West African countries [27,29,30,31,32]. The global similarity observed allowed the use of the VECTRI model to predict future malaria transmission.

**Figure 5:**
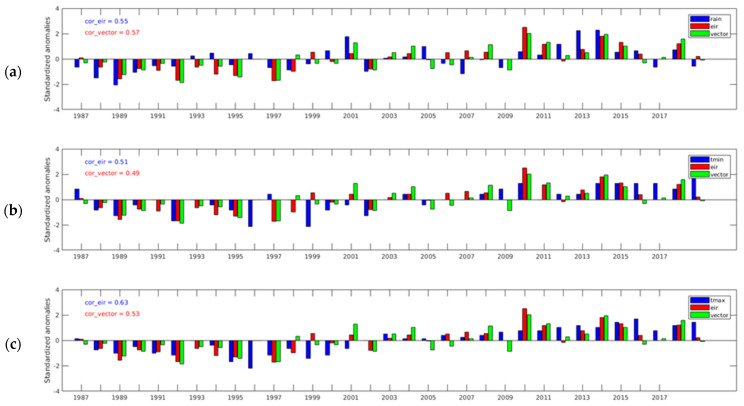
Anomalies of VECTRI-simulated EIR and vector density with (**a**) rainfall, (**b**) minimum temperature, and (**c**) maximum temperature in Tiassalé.

Figure 5 shows anomalies between the rainfall, temperature simulated EIR, and vector density in Tiassalé at a yearly scale. The EIR (red color) and vector density (green color) follow generally the same trends as T_min_ and T_max_ (deficit or surplus years) and show variability in precipitation for some years. The trends in EIR and vector density were in accordance with the trends observed in rainfall (Figure 5a). Before the year 2000, the rainfall was in deficit (decrease) and thereafter was in surplus (increase). The EIR correlation with T_max_ (0.63) was the highest, followed by the EIR correlation with rainfall (0.55), which was greater than that of T_min_ (0.51). EIR followed the trend in rainfall but exhibited interannual variability. However, these results were obtained without the non-climatic factors being included in the model and were integrated with the same population density. Previous studies have shown, in Emena (the forest zone of Ghana), that malaria transmission is predominantly controlled by rainfall [55]. This correlation is lower than it is in the Sahel regions, where malaria is more rainfall-driven. The maximum and minimum temperature surpluses are in accordance with the EIR and vector density (Figure 5b,c). These results confirm the known role of temperature in malaria transmission [22,29,59]. In Tiassalé, the dynamics of malaria patterns are controlled by the interaction between climate variables and the ecosystem (river, swamp, and amorphous soil). The EIR and vector density revealed an upward trend from 2003, which can be attributed to an increase in rainfall. Rainfall is a key factor that determines the abundance of mosquitoes and the length of the malaria transmission season [29,59,60,61].

In addition, daily life patterns, the location of homes relative to mosquito breeding sites, the type of materials used to build, and the structure of houses are key determinants of malaria transmission [62]. Thus, it is imperative to incorporate into the VECTRI model the permanent features of each area, such as rivers, lakes, and dams. For example, in Cameroon, the seasonality of malaria closely follows that of rainfall, with a lag of 1 to 2 months in locations far from permanent water bodies [21]. However, in locations close to permanent water sources, the seasonality of malaria is year-round, as in Tiassalé.

### 3.3. Relationship between Observed Meteorological Parameters and Malaria Transmission Patterns Derived from VECTRI

**Figure 6:**
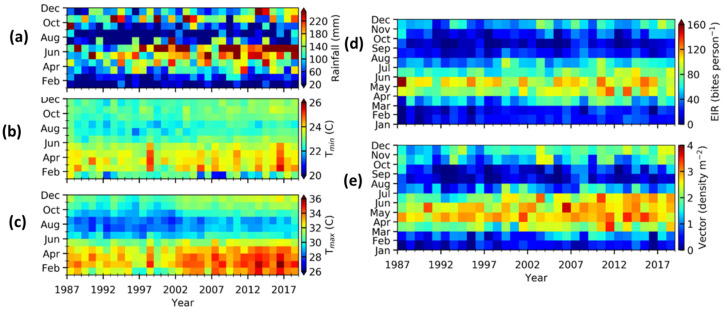
A Comparison of rainfall, temperature, and VECTRI-simulated EIR and vector density for Tiassalé over the period 1987 to 2019. (**a**–**c**) represent respectively the rainfall, T_min_ and T_max_. The malaria transmission indicators obtained by VECTRI model are EIR (**d**) and Vector density (**e**).

Figure 6 illustrates the monthly variability and relationship between meteorological parameters (temperature and rainfall) and malaria transmission patterns from 1987 to 2019.

**Figure 7:**
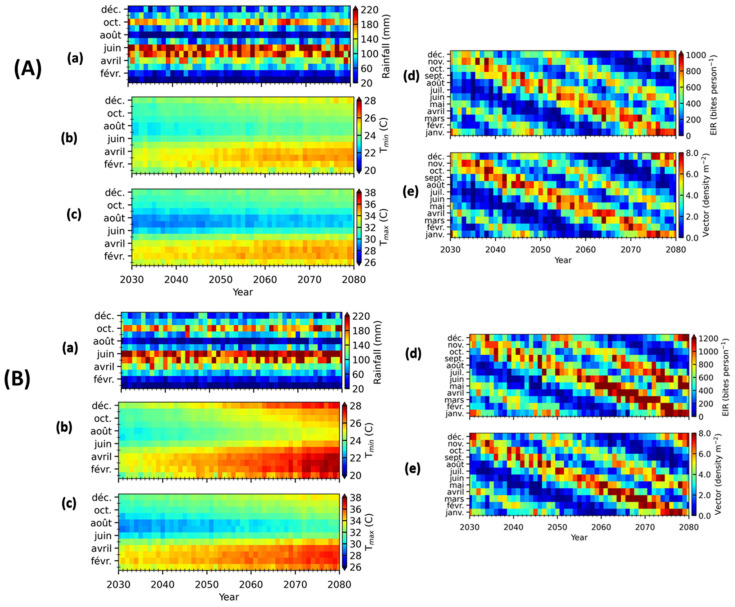
A comparison of rainfall, temperature, and VECTRI-simulated EIR and vector density for Tiassalé over the future period 2030 to 2080 under the RCP4.5 (**A**) and RCP8.5 (**B**) scenarios. For each subfigures, (**a**–**c**) represent the rainfall, T_min_ and T_max_ respectively. The malaria transmission indicators obtained by VECTRI model are EIR (**d**) and Vector density (**e**).

We observed a general increase in malaria transmission (the EIR and vector density) from March to July of every year (Figure 6d,e). The increasing trend was also observed in temperature and rainfall (Figure 7a–c). The high-temperature period overlaps with both the higher vector density and the EIR, with a time lag of one month. These results are within the range of observed malaria cases with high transmission all the year, except for the months of September and October.

During the dry season (July to October and December to February), the values of EIR and vector density decrease (Figure 6a,d,e). The upper and lower limits of temperature are within the range that supports malaria transmission. Of note, the life cycle of the vector is optimal under the temperatures between 28 °C and 32 °C [15,59,63]. However, temperatures exceeding 35 °C are detrimental to the growth and survival of mosquitoes [64]. Within this period, the EIR and vector density values increase when the temperature remains within the range of 22–26 °C (T_min_) or above 30 °C (T_max_) and rainfall values decrease below 10 mm·month^−1^. We found that, on average, the EIR and vector density are higher during April to June compared to over the period of the year.

The above result confirms that the VECTRI model is able to account for the relationship between rainfall, temperature, and malaria transmission.

### 3.4. Malaria Prediction

Figure 7 shows the changes in the EIR in Tiassalé for the period 2030 to 2080. For the scenario RCP4.5, the monthly EIR varies between 0.48 and 908 bites per person from 2030 to 2080. The number of bites or the EIR for the scenario RCP8.5 is very high, between 1.74 and 1131.71 bites per person per month, compared to RCP4.5. In addition, it should be emphasized that the VECTRI model was also able to account for the significant impact of temperature in malaria transmission dynamics. For instance, despite the fact that, in scenarios RCP4.5 and RCP8.5, almost similar rainfall patterns can be observed (Figure 7A(a),B(a)), the VECTRI-simulated EIR and vector density show lower values at RCP4.5 relative to RCP8.5 (Figure 7A(d,e),B(d,e)). This observation is significantly due to the lower temperatures recorded at RCP4.5 relative to RCP8.5. Malaria transmission varies from one year to the next. For RCP8.5, low transmissions were observed from March to May and from August to September for the years 2050 and 2080, respectively. The same increasing trend of EIR was observed for the study period 1987 to 2019, which suggests that malaria will still be an important cause of mortality in Tiassalé in the coming year. This trend is similar to what was observed in East Africa, where malaria transmission tended to increase between 2008 and 2052 under the RCP8.5 scenario [52]. In addition to predicting the malaria peak months in Tiassale, the VECTRI model was able to predict EIR values for the coming decade.

A previous study predicted an increase in malaria transmission, with an additional 51.3 million people to be at risk of malaria in West Africa by 2050 [22]. Furthermore, hotspots of seasonal malaria transmission suitability in both climate scenarios (RCP4.5 and RCP8.5) are predicted to either continue to concentrate or shift both northward and southward into the highlands of Ethiopia and Southern Africa toward 2050 [22]. Despite malaria awareness campaigns in Côte d’Ivoire, the number of malaria cases remains high in Tiassalé. In addition, the simulated EIR and density vector were important, showing that there is a need to evaluate the potential of ongoing malaria control interventions in the country. There is a possibility of using the VECTRI model to provide an advance warning of malaria transmission and call for anticipating malaria control actions.

### 3.5. Study Limitations

There are two main limitations to this work, namely, the unavailability of meteorological station data in the study city and the lack of clinical data on malaria morbidity in all the town’s health centers over a sustained period of at least 10 years. The use of satellite data (ARC2 and ERA5) for the city of Tiassalé remained the only possible option for characterizing the climatic dynamics of the study area, after obtaining a satisfactory correlation between ARC2 and ERA5 data from four other cities surrounding Tiassalé and data from meteorological stations in these cities. For malaria morbidity data, only one health center had a complete set of consultation data for ten consecutive years. Therefore, these data were used to assess the prevalence of malaria as a cause of consultation in this health center. This morbidity indicator may therefore not represent the reality of all malaria morbidity in Tiassalé. These two weaknesses respecting meteorological and health data may explain the low correlations observed between them and between malaria morbidity and the outputs of the VECTRI model, which also uses satellite data. However, the study has the merit of outlining ways of circumventing the difficulties inherent in building scientific evidence on the links between climate and malaria transmission in African areas suffering for the most part from the same difficulties of access to quality data. The study also enabled the VECTRI model to be tested in an endemic area of annual high malaria transmission and to be used to predict future transmission. It is likely that transmission within Tiassalé may be different from the model results due to some effects not accounted for in the VECTRI model, such as permanent water bodies that can sustain transmission during the dry season.

## 4. Conclusions

Malaria transmission in Tiassalé is annual, with peaks from May to July and September to November. December to February and August are the driest periods of the year. Average temperatures are highest from December to May. The monthly EIR values obtained from the VECTRI model fit well overall with observed malaria cases, rainfall, and temperatures from 2010 to 2019. The VECTRI model predicts an increase in malaria transmission (EIR and vector density) between 2030 and 2080 and calls for a strengthening of the national malaria control strategy to combat this trend.

## Figures and Tables

**Figure A1:**
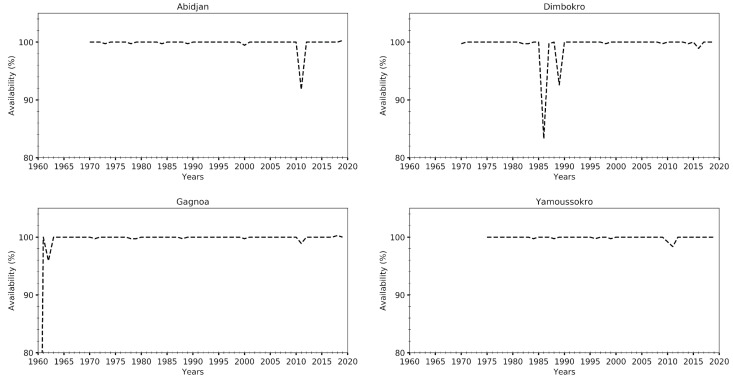
Completeness of the rainfall and temperature (minimum and maximum) observational data from the four closest stations to Tiassalé.

**Figure A2:**
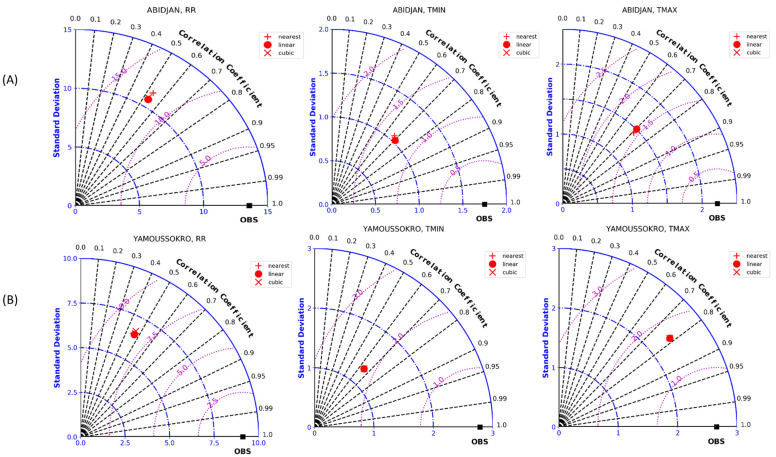

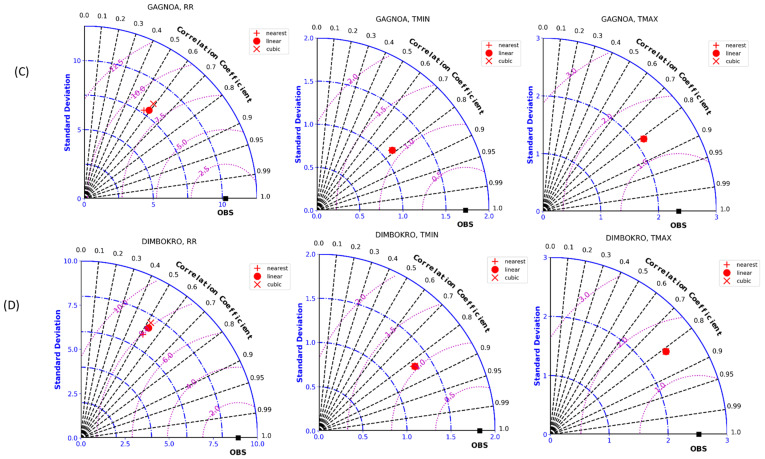
Taylor diagrams illustrating comparisons of daily rainfall (ARC2) and minimum and maximum temperature (ERA5) datasets as a reference for the observational meteorological data obtained from the closest four stations to Tiassalé, namely, (**A**) Abidjan, (**B**) Yamoussoukro, (**C**) Gagnoa, and (**D**) Dimbokro. The dotted lines indicate the correlation coefficients; the standard deviations are shown in blue and the root mean square errors in magenta. In the **top** row, “RR” means rainfall.

**Figure A3:**
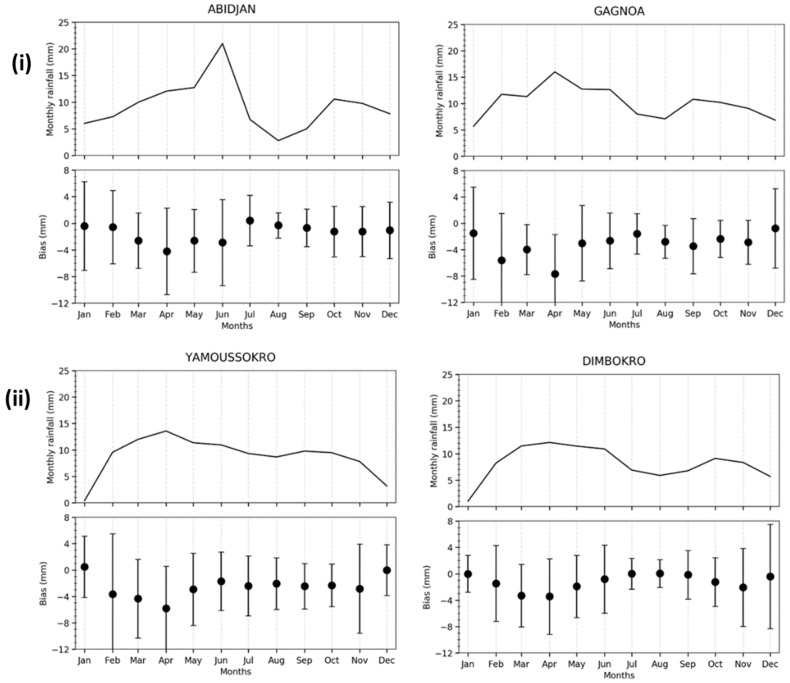
(**i**) Monthly averages of precipitation from the four stations closest to Tiassalé (Abidjan, Yamoussoukro, Gagnoa, and Dimbokro). (**ii**) Error bars, where the medians of the biases (ARC—OBS) are represented in the middle, and the 25th and 75th percentiles are represented by the bars.

**Figure A7:**
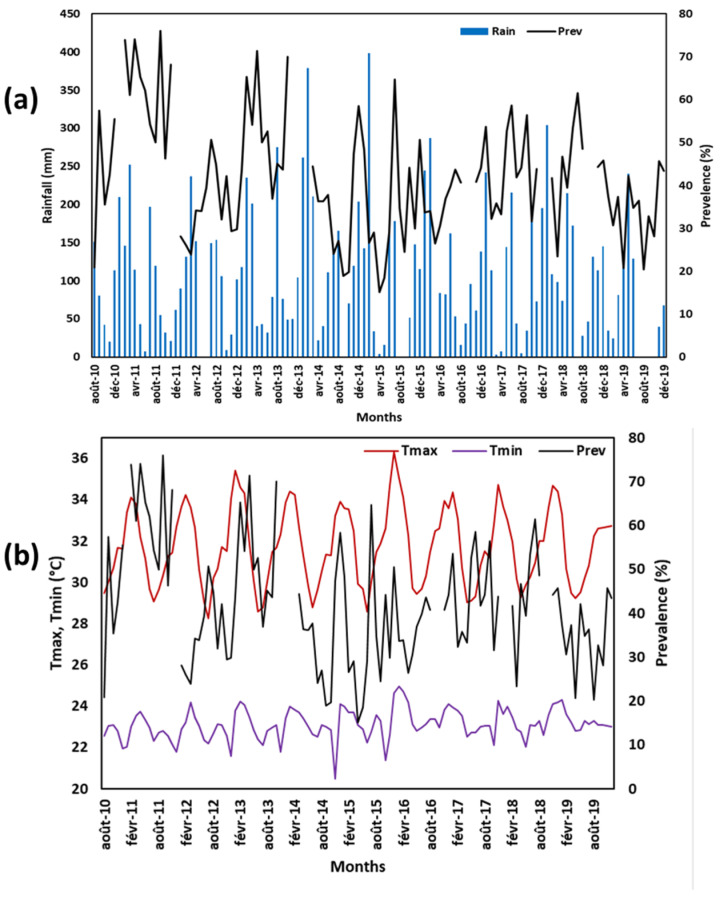
Comparison of monthly prevalence with (**a**) rainfall and (**b**) T_max_ and T_min_ data for Tiassalé over the period 2010 to 2019.

## Data Availability

Historical health data on the number of malaria cases were obtained from hospitals in Tiassalé. ARC2 data available at: https://www.ngdc.noaa.gov/docucomp/page?xml=NOAA/NWS/NCEP/CPC/iso/xml/Daily-ARC2-Africa.xml&view=getDataView&header=none. ERA 5 data downloaded at: https://cds.climate.copernicus.eu/cdsapp#!/dataset/reanalysis-era5-single-levels?tab=overview), accessed on 11 August 2020.

## Appendix A. Assessing the Suitability of ARC2 and ERA5 Data

We compared data from the satellite with data from four weather stations located around Tiassalé. First, we carried out a quality control to assess the completeness of the four stations’ data and their consistency over the period from 1960 to 2019 (see Figure A1). We then used Taylor diagrams to summarize the similarities and evaluate the consistencies between observational data from these weather stations (Yamoussokro, 1975–2019; Abidjan, 1970–2019; Gagnoa, 1960–2019; and Dimbokro, 1970–2019) [46] and the estimated precipitation data from ARC2 and the temperature data (T_min_ and T_max_) from ERA5 (see Figure A2).

Figure A1 shows how the quality control was carried out to check the completeness of the SODEXAM data and to assess their consistency. We found that more than 80% of the daily weather data existed for the four (4) stations (Abidjan, Yamoussoukro, Gagnoa, and Dimbokro) close to Tiassalé. We then considered the level of confidence acceptable for the time series data.

The Taylor diagram summarizes the similarity between two data (estimations and observations) by showing a polar graph correlation coefficient (gauging similarity in pattern between the estimated and observed fields). The root mean square error (RMSE) in the estimated field is proportional to the distance from the point identified as “observed”, and standard deviations of the estimated pattern are proportional to the radial distance from the origin. Taylor diagrams are commonly used for multi-model comparison [65]. The centered pattern or the root mean square error (RMSE) difference is the mean-removed RMSE difference, calculated as follows:

(A1) $$RMSE=\frac{1}{n}\sum_{n=1}^{N}{\left[(f_{n}-f)-\left(r_{n}-r\right)\right]}^{2}$$

where RMS is the root mean square error, *f_n_* is the time series of the model parameter that is being tested, and *r_n_* is the time series of the observation data. The correlation coefficient is related by:

(A2) $$x=\frac{\frac{1}{n}\sum_{n=1}^{N}\left(f_{n}-f\right)\left(r_{n}-r\right)}{\sigma_{f}\sigma_{r}}$$

where *x* is the correlation coefficient and *σ_f_* and *σ_r_* are the standard deviations of the model and observation, respectively.

Figure A2 shows the Taylor diagrams for the four stations, namely, Abidjan, Yamoussoukro, Gagnoa, and Dimbokro. Overall, the three interpolation (bilinear, cubic, and nearest) methods used to extract ARC2 data at different station locations gave the same information for rainfall and temperature. Here, we chose the nearest method for this study. Over the four stations, the T_min_ and T_max_ values showed a high correlation and the lowest RMSE (Figure A2). ERA5 reproduces well over the T_min_ and T_max_. We conclude that these data can be used for Tiassalé.

Regarding rainfall, the correlation coefficient is around 0.5. This explains why barely 50% of the ARC information was correlated with the observed data. Shown in purple, the root mean square error is around 8, and, shown in blue, the standard deviation is very high (around 7.5). ARC can keep up with the daily variability of rainfall even if the correlation is average.

When assessing the monthly variability of precipitation, the average was obtained by cumulating the rainfall over the number of rainy days for each month. This allowed the peak of the rainy season to be determined from the observed data. The graphs in Figure A3(i) show two peaks for the four stations, the first peak (between March and June) and the second (between September and October).

Figure A3(ii) shows the bias, the difference between the precipitation from the ARC data and the observed data for each month. ARC is able to reproduce the monthly variability with some biases. We decided to use ARC2 rainfall data for Tiassalé.

Overall, the three interpolation (bilinear, cubic, and nearest) methods used to extract the data from ARC2 and ERA5 at different stations provided the same information for the rainfall and the temperature (Figure A2). The daily T_min_ and T_max_ values from the four stations revealed a high correlation and the lowest root mean square error (RMSE) (Figure A2). We observed a good agreement between T_min_ and T_max_ values derived from ERA5 compared to those from the local weather station. ARC2 was globally able to produce the daily variability of observed rainfall with a correlation coefficient varying from 0.5 to 0.6. The correlation coefficient for monthly data was higher than for daily data (Figure A3). We then used ERA5 data (temperatures) and ARC2 data (rainfall) covering the period from 1987 to 2019 for the study.

Figure A4 shows the Taylor diagrams of the simulated model and the observed crude values for Tiassalé used to select the best model among the 14 CORDEX models for scenario 8.5. The multi-model means (EMM) of the CORDEX-Africa simulations (MEAN) and the BWRF model were close to the observations and are better than other data.

For this study, we then used EMM or MEAN model data as input data (daily precipitation, T_min_, and T_max_) for VECTRI to perform future simulations.

Figure A5 shows the list of 14 models of CORDEX-AFRICA data.

Figure A6 shows the output files after running VECTRI.

## Appendix B. Statistical Analysis

**Table A1:** Summary statistics.

Variable	Observations	Obs. with Missing Data	Obs. without Missing Data	Minimum	Maximum	Mean	Std. Deviation
PREV	102	0	102	0.000	76.000	41.444	14.419
EIR	102	0	102	1.160	130.060	53.850	33.716
Vector	102	0	102	0.005	3.518	1.605	1.012
Rain	102	0	102	0.000	398.600	109.249	89.238
T_max_	102	0	102	28.269	36.313	31.572	1.885
T_min_	102	0	102	20.490	24.963	23.111	0.713

**Table A2:** Correlation matrix (Pearson (n)).

Variables	Malaria Cases	Rain	T_max_	T_min_	EIR	Vector
CaseM	**1**	−0.071	0.031	0.151	0.042	−0.086
Rain	−0.071	**1**	−0.041	**0.322**	0.041	0.151
T_max_	0.031	−0.041	**1**	**0.524**	**−0.318**	**−0.279**
T_min_	0.151	**0.322**	**0.524**	**1**	−0.130	−0.159
EIR	0.042	0.041	**−0.318**	−0.130	**1**	**0.854**
Vector	−0.086	0.151	**−0.279**	−0.159	**0.854**	**1**

Values in bold are different from 0 with a significance level.
